# General Health and Resilience of Breast Cancer Patients: The Mediator Role of Affective Well-Being

**DOI:** 10.3390/ijerph19095398

**Published:** 2022-04-28

**Authors:** M. Victoria Cerezo, Ana Álvarez-Olmo, Pilar Rueda

**Affiliations:** Faculty of Psychology, University of Malaga, Teatinos Campus s/n, 29071 Malaga, Spain; anaalvarezolmo@gmail.com (A.Á.-O.); pilarrueda@uma.es (P.R.)

**Keywords:** breast cancer, affective well-being, resilience, general health, mediation model

## Abstract

A considerable percentage of breast cancer patients present adequate psychological adjustment and do not become distressed after a breast cancer diagnosis, or, if they do, they manage to recover quickly, which is reflected in their general health. This study aims to determine the role of some psychological mechanisms that affect psycho-oncological adjustment, specifically, resilience and well-being, in a sample of 109 breast cancer patients. For this purpose, participants completed questionnaires on general health, resilience, and well-being (life satisfaction and affect). Correlation analyses and a multiple mediation model were carried out. The results revealed that Pearson correlations between all variables showed strong associations between general health scores and positive and negative affect scores, and moderate associations with life satisfaction and resilience scores. Furthermore; in the mediation model, the total percentage of variance explained by the overall model was 55% (*R*^2^ = 0.55), where resilience was associated with positive and negative affect, and that influenced general health. These results show that affective well-being is especially relevant in breast cancer patients in terms of its mediating role in resilience, making it clear that an appropriate intervention focused on managing patients’ affective status can have a favorable impact on their overall health.

## 1. Introduction

Cancer is the process in which the body’s cells grow uncontrollably. In women, it is the main cause of cancer death [1]. Specifically, breast cancer is one of the most common cancers, with 2.6 million cases diagnosed, which are expected to increase by 60% by 2030 [2]. In Spain, it is the main deadly cancer in women, with similar percentages in several countries around the world [3]. Age, genetic predisposition, familial cancer, hormonal factors, benign proliferations, and environmental factors are among the multiple factors that cause breast cancer; Nonetheless, in 50% of cases diagnosed, the cause is unknown [4,5].

Breast cancer deteriorates patients’ health and quality of life, due to its consequences in different life domains such as physical, emotional, social, and economic [6,7,8,9,10,11]. Around 40% of cancer patients present comorbid anxiety/mood spectrum disorders [12]. Concretely, the prevalence rate of anxiety disorder in breast cancer patients is 41.9% and is even higher in Mediterranean countries [13]. These symptoms are associated with other disorders affecting patients’ health, such as worries and insomnia [14]. On the bright side of these data, this means that there is a considerable percentage of women who present good psychological adjustment and do not become distressed after a breast cancer diagnosis, or if they do, they manage to recover quickly. This fact indicates the recovery capacity or resilience manifested by these women [15,16]. There is growing interest in clarifying which positive variables can buffer the emotional distress caused by cancer in these women [17]. In this regard, to date, some mediation studies consider variables such as social support [18,19] or resilience [20,21] to act as mediators for positive coping with cancer. For example, recently, Zhou et al. [21] confirmed the mediating role of resilience in coping styles, perceived social support, and health-related quality of life, but they did not consider well-being or general health.

In this sense, well-being has been a variable of great interest concerning breast cancer patients [22,23,24,25]. Well-being has been defined as the evaluation of our life as a whole, differentiating between two components: cognitive and affective. The cognitive part refers to satisfaction with life: the cognitive perceived discrepancy between aspirations and achievements [26]. The affective aspect refers to the balance of emotions and moods frequently experienced by the person, making the assessment of positive and negative affect independently [26,27]. Affect is defined as the person’s emotional predisposition. That is, affect is the substrate of emotions and feelings [28]. Thus, negative affect comprises the whole set of emotions related to sadness, apathy, boredom, frustration, anger, etc. In contrast, positive affect refers to the person’s predisposition to experience positive emotions such as engagement, happiness, calmness, or interest [26,28,29]. Some studies report that satisfaction with life (the cognitive aspect of well-being) of breast cancer patients is lower than that of other cancer patients [12,22,30], although, in general, they score at a medium level [22,24]. Regarding affective well-being, it has been shown that scores on positive and negative affect are related to resilience [31], although more studies are needed to show whether positive affect can influence the general health status of breast cancer patients. To our knowledge, few studies have explored the role of affect: there is only one recent study, on perceived social support, that considers affective experiences as possible mediators between social support and satisfaction with life [19]. 

The presence or absence of psychiatric symptoms, for example, depressive or anxious symptoms, should be taken into account when referring to well-being. Depressive or anxious symptoms may be present in patients at levels low enough to be undiagnosable as a disorder but high enough to affect psychological well-being. The absence of this set of symptoms, together with the absence of somatic symptoms, social dysfunction, and insomnia is called general health [32]. Apart from their physical health, it is important to pay attention to the general health of breast cancer patients with regard to the impact that the illness and its consequences have on all aspects of their lives [12,14].

In addition to the level of affect (or affective well-being) and the impact that breast cancer has on general health, the literature has shown that an essential variable in recovery after a traumatic event of any kind is resilience [15,33]. Resilience is a personality variable that, despite its constant presence in human beings, has not been defined and studied in detail until the rise of positive psychology in the 1990s [34]. Resilience is the ability to overcome a negative or painful experience and turn it into a source of learning and growth [34,35]. Scientists working with cancer patients, specifically with breast cancer, corroborate that women who present higher levels of resilience are those who cope with the disease more adaptively (maintaining higher levels of social functioning) [16] and with less anxious–depressive symptomatology [12,17,20,23,31,36,37]. In breast cancer patients, resilience has been found to be associated with greater positive affect and well-being [17,31] and minor negative affect [17,31,38]. Guil et al. [20] pointed out that resilience may be mediated by emotional intelligence. At this point, we propose our research questions regarding the relationship between well-being, satisfaction with life, resilience, and general health: Are well-being, satisfaction with life, and positive and negative affect related to the patient’s capacity for resilience? Do the levels of well-being experienced by patients mediate their resilience shown during the illness? Does this relationship influence the patients’ general health? This study intends to answer these questions. Thus, the aims of this study are, first, to know how general health is related to resilience and well-being, both cognitive and affective; secondly, to clarify the role of well-being as a possible mediator of resilience and general health in breast cancer patients.

## 2. Methods

### 2.1. Study Design and Procedure

This study uses a cross-sectional design. Convenience sampling was used to recruit the participants. The study and questionnaire were designed in October 2021. Staff from several healthcare centers contacted the participants via email, asking them to participate by completing an online questionnaire (via Google Forms platform). All participants were volunteers; there were no incentives. Informed consent was given and signed by all of the participants, their anonymity was protected, and the data were only used for research purposes. The data collection started in November 2021. The questionnaire was designed so that all the questions were mandatory, and there were no missing data. Informed consent to participate in this study was required, and a statement about the use of the data collected and confidentiality was included at the beginning of the questionnaire. No identifiable personal information was stored. In January 2022, the data collection was completed, ready to perform the analysis and obtain the results. Finally, the manuscript was accomplished in March 2022.

### 2.2. Participants

The sample was composed of 109 Spanish women with breast cancer. Their ages ranged from 31 to 75 years (*M* = 52.71, *SD* = 9.19). The mean time since the diagnosis ranged from several months to 23 years (*M* = 6.01, *SD* = 5.32). About 27% of the participants were from the north of Spain and 72.2% were from the south. These and other socio-demographic characteristics are shown in Table 1.

### 2.3. Instruments

Sociodemographic data regarding place of residence, age, marital status, number of children, educational level, and occupation, and clinical history data regarding the date of cancer diagnosis, time, stage, and axillary dissection were recruited using multiple-choice questions. 

General health was measured using the Goldberg General Health Questionnaire (GHQ-28) [32] in its Spanish version [39]. It comprises 28 items distributed in four subscales of seven items that measure somatic symptoms, anxiety/insomnia, social dysfunction, and major depression. Higher total scores in each subscale are indicative of lower health (general or referring to the subscale). According to Nourbala et al. [40], the threshold value of the GHQ-28 is 23; thus, scores below 22 were indicative of a healthy status, and ≥23 show an unhealthy status. For this sample, Cronbach’s alpha for somatic symptoms was 0.87; for anxiety and insomnia, it was 0.93; for social dysfunction, it was 0.80; and for major depression, it was 0.91, respectively. Cronbach’s alpha for the total score was 0.94 in this sample.

Satisfaction with life (cognitive well-being) was measured via the Satisfaction with Life Scale (SWLS) [26], in its Spanish version, validated in Spanish breast cancer patients [22]. This instrument comprises five items rated on a seven-point Likert-type scale (1 = strongly disagree; 7 = strongly agree), with higher scores indicating greater satisfaction with life. Cronbach’s alpha in the present sample was 0.89.

The affective component of well-being was measured using the Negative and Positive Affect Scale (NAPAS) [41]. This instrument comprises two subscales with six items for positive and another six items for negative affect, rated on a five-point Likert-type scale (1 = never; 5 = always). Higher scores are indicative of higher levels of positive or negative affect, respectively. For this sample, Cronbach’s alpha for positive affect was 0.91 and for negative affect, it was 0.92.

To measure resilience, we used the brief version of the Connor-Davidson Resilience Scale (CD-RISC 10) [42], based on the CD-RISC 25 [43], in its Spanish version validated in Spanish breast cancer patients [31]. This instrument comprises 10 items rated on a five-point Likert-type scale (0 = not true at all; 4 = true nearly all the time). Higher scores indicate greater resilience. Cronbach’s alpha in the present sample was 0.95.

### 2.4. Data Analysis

For data analysis, the statistical software SPSS v.25 (IBM^®^ SPSS^®^ Statistics license assigned to University of Malaga, Spain) was used. Preliminary analyses were carried out to compute descriptive statistics about sociodemographic variables and internal consistencies for the instruments, using Cronbach’s alpha for this sample. Hence, we computed Pearson correlation coefficients between all the variables’ scores, considering (following Cohen’s criterion) coefficients of |0.10| as small, of |0.30| as moderate, and of |0.50| or high as strong correlations.

Finally, a multiple mediation analysis was performed to test whether well-being mediated the relationship between the total score of general health and resilience after controlling for the influence of age. The confidence intervals (CI) were calculated through the 10,000 estimates of the indirect effect bootstrap samples, considering an indirect effect statistically significant when the 95% CI did not include zero.

## 3. Results

Firstly, descriptive statistics and correlations were calculated. Adequate correlations between all of the variables were calculated. On one hand, none of the sociodemographic variables correlated significantly with well-being, except for the number of children, which generated a positive and significative correlation with satisfaction with life (Pearson coefficient = 0.23; *p* = 0.016). On the other hand, results regarding psychological variables showed strong associations between scores of general health (each subscale and total score) and scores of positive and negative affect, and moderate ones with scores of satisfaction with life and resilience. Particularly, considering that high general health scores are indicative of worse general health, the relationship of the scores with positive affect, satisfaction with life, and resilience are inverse, whereas with negative affect, it was direct. Resilience was also found to be strongly related to affective well-being: directly with positive affect and indirectly with negative affect, but it did not correlate with satisfaction with life. Age was not significantly related to any variable. Table 2 shows the correlations between the variables.

**Figure 1:**
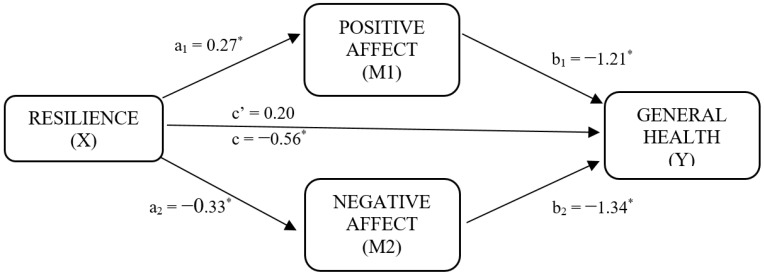
Diagram of the multiple mediation model: direct and indirect effects among resilience, positive and negative affect (affective well-being), and general health. Note: * Significant at *p* < 0.001 level. a_1_: direct effect of positive affect on resilience; a_2_: direct effect of negative affect on resilience; b_2_: direct effect positive affect on general health; b_2_: direct effect negative affect on general health; c’: direct effect of resilience on general health; c: direct effect of total effect of resilience on general health.

Once the variables were explored, mediation analyses were carried out with the PROCESS macro for IBM^®^ SPSS^®^ Statistics (license assigned to University of Malaga, Spain) [44], considering the role of affective well-being as a mediator in resilience, with outcomes in general health. Age was not included in the mediating model as a covariate because it was not correlated with the variables involved. In addition, we did not perform the analysis with cognitive well-being because the correlations were nonsignificant. Figure 1 summarizes the diagram of the mediating model, and Table 3 shows the results.

Concretely, the mediation analysis included resilience as an independent variable, positive and negative affect as mediating variables, and general health as a dependent variable. Results based on 10,000 bootstrap samples indicated that whereas the total effect of resilience on general health was significant (c = −0.56, *SE* = 0.13, *p* < 0.001), the direct effect was not (c’ = 0.20, *SE* = 0.12, *p* = 0.11), suggesting a total mediation. Furthermore, as seen in Table 3, the total percentage of variance explained by the overall model was 55% (*R*^2^ = 0.55), and this result was statistically significant.

Regarding the statistically significant direct effects obtained, positive affect (b_1_) was negatively related to perceived health, suggesting that women with breast cancer with higher levels of positive affect showed better general health. In addition, negative affect (b_2_) was positively and statistically significantly associated with perceived health, indicating that women with greater negative affect would obtain worse scores in general health. The results of the mediation analysis also revealed that the two contrasted indirect effects were statistically significant (95% CI; see Table 3), both exerting a positive influence on general health. Thus, indirect effect 1 (a_1_b_1_) indicated that resilience increases the levels of positive affect, and positive affect increases general health in women with breast cancer (B = −0.32; BootSE = 0.07; 95% CI [−0.48, −0.19]). The indirect effect 2 (a_2_b_2_) suggested that high resilience values reduce negative affect, thus increasing the participants’ general health (B = −0.44; BootSE = 0.09; 95% CI [−0.64, −0.26]). 

To determine which indirect effect presented greater statistical weight, a contrast analysis of the two significant indirect effects was performed. Considering the sign of the coefficients, the analyses indicated that there were no statistically significant differences between the weight of the two.

## 4. Discussion

In this study, we explored well-being (both cognitive and affective), resilience, and general health in breast cancer patients. To know the psychological mechanisms underlying this illness is very important with regard to its prevalence and as a resource of support for the patients and their recovery. For this purpose, firstly, the relationship between cognitive and affective well-being, resilience, and general health was explored. Pearson’s correlations between all the variables showed strong associations between scores of general health (total score) and scores of positive and negative affect, and moderate associations with scores of satisfaction with life and resilience. This means that patients with lower general health also showed lower well-being, in line with previous research that has already shown low levels of well-being in patients with poor levels of adjustment to cancer [11,12,25,30]. Furthermore, these results point to a strong association between resilience and positive and negative affect, as in previous research [31]. In contrast, our data failed to show a significant correlation between resilience and satisfaction with life [12,31], which indicates that further research in this regard is needed.

To explore a possible mediating role of affect between resilience and general health, a mediational analysis was carried out. This model was significant. Focusing on the direct effects of resilience, a positive and significant predictive association of positive affect and an inverse predictive association of negative affect were found, indicating that affective well-being mediates resilience in the general health of breast cancer patients. These results are in line with previous research regarding affective well-being that showed it to be positively associated with personal growth after experiencing adverse situations [31,45]. To our knowledge, no studies to date have shown a mediating role of affective well-being on resilience, as the most recent studies focused on emotional intelligence and social support [18,19,20,21].

Thus, resilience was associated with positive affect and negative affect, separately, which, in turn, influenced general health. Resilience has a greater effect on general health through affective well-being. These results suggest that breast cancer patients with a higher level of resilience were more likely to report a higher level of positive affect and a lower level of negative affect, influencing their general health. 

To gain knowledge about this mediating mechanism is important, not only to understand the psychological processes underlying breast cancer but also for the design of psychological interventions. If treatment aims to reduce the symptoms of psychological maladjustment, that is, to increase general health, we know that the intervention must be oriented to increasing the patient’s affective well-being (not to decreasing their negative affect), in the same way that interventions focused on resilience will favor general health through affect. Previous psychological interventions with a positive psychology approach have already demonstrated their efficacy [46,47,48] because they have worked on improving resilience, among other aspects of psychological functioning. Thus, it has been shown that psychological support during the treatment is of great importance in the recovery from the disease, and the researchers insist that it should also be provided to survivors once the treatment is over [14,15]. Both during treatment and after, when patients become survivors, it is important to foster resilience in order to avoid psychological distress and general decline [46]. Once the most aggressive treatments have been completed, it is still important to accompany the patient until she has recovered her routines and habits prior to diagnosis. In short, fostering resilience is of vital importance to facilitate and promote improvement in patients’ general health.

This study has some limitations, for example, the use of self-report measures in data collection and collecting the data online. Although self-report and online questionnaires give the patients more intimacy, they also allow social desirability to affect the answers as well as distractions while completing the questionnaires. In addition, the causal interpretation is not possible because this is a cross-sectional study that used a convenience sample. 

Despite these limitations, it can be stated that affective well-being is especially relevant in breast cancer patients in terms of its mediating role with resilience, clearly showing that an appropriate intervention focused on the management of patients’ affective state can have a favorable impact on their general health.

## 5. Conclusions

This study shows that affective well-being is especially relevant in breast cancer patients in terms of its mediating role with resilience, because resilience has a greater effect on overall health through affective well-being. Consequently, if psychological intervention focuses on managing patients’ affective state and resilience, it is possible that it will have a favorable impact on their overall health, promoting better coping with cancer. Therefore, now that we know this, we encourage psycho-oncologists to design psychological interventions aimed at increasing the patient’s affective well-being.

## Figures and Tables

**Table 1:** Sample characteristics (*N* = 109).

Variables	*N*	%
Age		
<50 years	43	39.4
>50 years	66	60.6
Marital status		
Single	25	23.0
Married	70	64.2
Divorced	12	11.0
Widowed	2	1.8
Educational level		
Primary	12	11.0
Secondary	13	11.9
Other non-university	31	28.5
University	53	48.6
Occupation		
Home-keeper	16	14.7
Employed	78	71.6
Unemployed	7	6.4
Retired	8	7.3
Number of children		
0	27	24.8
1	24	22.0
2	52	47.7
>2	6	5.5
Breast cancer stage		
0	3	2.8
I	19	17.4
II	52	47.7
III	33	30.8
IV	2	1.8
Axillary dissection		
No	39	35.8
Yes	70	64.2
Time since diagnosis (years)		
<2	31	28.5
2–5	36	33.0
>5	38.5	38.5

**Table 2:** Correlations between variables studied.

Variables	General Health (Total Score)	1 (SS)	2 (A&I)	3 (SDY)	4 (MA)	5 (PA)	6 (NA)	7 (SWL)
1 Somatic symptoms (SS)	0.76 *							
2 Anxiety and insomnia (A&I)	0.88 *	0.56 *						
3 Social dysfunction (SDY)	0.77 *	0.47 *	0.61 *					
4 Major depression (MA)	0.75 *	0.35 *	0.55 *	0.47 *				
5 Positive affect (PA)	−0.55 *	−0.30 *	−0.46 *	−0.43 *	−0.56 *			
6 Negative affect (NA)	0.65 *	0.36 *	0.59 *	0.54 *	0.56 *	−0.35 *		
7 Satisfaction with life (SWL)	−0.28 *	−0.15	−0.23 **	−0.20 **	−0.38*	0.40 *	−0.20 **	
8 Resilience	−0.37 *	−0.19 **	−0.33 *	−0.35 *	−0.40 *	0.54 *	−0.52 *	0.12

Note: * *p* < 0.001. ** *p* < 0.05.

**Table 3:** Mediation model with model summary, direct effect, and indirect effect.

**Model Summary**		***R* ^2^**	**MSE**	**F**	**df_1_**	**df_2_**	***p***
Total effect model		0.17	187.28	10.60	2	106	<0.001
Positive affect on resilience		0.29	18.97	22.41	2	106	<0.001
Negative affect on resilience		0.30	2929	23.11	2	106	<0.001
General health on resilience		0.55	102.16	32.30	4	104	<0.001
**Direct effects**	**Path**	**Coef.**	***SE***	**T**	**95% CI**		
					***p***	**BootLL**	**BootUL**
Positive affect on resilience	a_1_	0.27	0.04	6.59	<0.001	0.19	0.34
Negative affect on resilience	a_2_	−0.33	0.05	−6.51	<0.001	−0.43	−0.23
Positive affect on general health	b_1_	−1.21	0.22	−5.39	<0.001	−1.65	−0.76
Negative affect on general health	b_2_	−1.32	0.17	7.42	<0.001	0.97	1.68
Resilience on general health	c’	0.20	0.12	1.60	0.11	−0.05	0.44
Total effect of resilience on general health	c	−0.56	0.13	−4.45	<0.001	−0.81	−0.31
**Indirect effects**			**Path**	**Effect**	**BootSE**	**BootLL**	**BootUL**
Total				−0.75	0.13	−1.02	−0.52
Resilience -> Positive affect -> General Health			a_1_b_1_	−0.32	0.07	−0.48	−0.19
Resilience -> Negative affect -> General health			a_2_b_2_	−0.44	0.10	−0.64	−0.27
C1				0.12	0.11	−0.10	0.34
